# Mechanism of Fatigue-Life Extension Due to Dynamic Strain Aging in Low-Carbon Steel at High Temperature

**DOI:** 10.3390/ma17184660

**Published:** 2024-09-23

**Authors:** Zheng Fang, Lu Wang, Fengyun Yu, Ying He, Zheng Wang

**Affiliations:** 1 School of Energy and Power Engineering, Dalian University of Technology, Dalian 116024, China; 2 School of Materials Science and Engineering, Dalian University of Technology, Dalian 116024, China

**Keywords:** EBSD, HR-EBSD, high-power XRD, prolonged fatigue life, low-carbon steel

## Abstract

An enhancement in fatigue life for ferrite–pearlite low-carbon steel (LCS) at high temperature (HT) has been discovered, where it increased from 190,873 cycles at room temperature (RT) to 10,000,000 cycles at 400 °C under the same stress conditions. To understand the mechanism behind this phenomenon, the evolution of microstructure and dislocation density during fatigue tests was comprehensively investigated. High-power X-ray diffraction (XRD) was employed to analyze the evolution of total dislocation density, while Electron Backscatter Diffraction (EBSD) and High-Resolution EBSD (HR-EBSD) were conducted to reveal the evolutions of kernel average misorientation (KAM), geometrically necessary dislocations (GND) and elastic strains. Results indicate that the enhancement was attributed to the dynamic strain aging (DSA) effect above the upper temperature limit, where serration and jerky flow disappeared but hindrance of dislocations persisted. Due to the DSA effect, periods of increase and decrease in the total dislocations were observed during HT fatigue tests, and the fraction of screw dislocations increased continuously, caused by viscous movement of the screw dislocations. Furthermore, the increased fraction of screw dislocations resulted in a lower energy configuration, reducing slip traces on sample surfaces and preventing fatigue-crack initiation.

## 1. Introduction

To date, the microstructure of low-carbon steels (LCS) has been investigated extensively due to the essential role of LCS in industry; also, their deformation mechanism is quite unique compared with other industrial metals because of the effect of Cottrell atmosphere at different temperatures, often referred to as the dynamic strain aging (DSA) effect. It has been pointed out that the tensile stress–strain curve of LCS has various shapes, namely smooth curves, serrated curves, and jerky flow [1]. As temperature increases, jerky flow can be observed first, usually at room temperature (RT); then the curve reshapes to serrated flow at about 100~300 °C, depending on content of interstitial atoms [2,3,4]. In the temperature range of 100~300 °C, mobile interstitial atoms interact dynamically with edge and screw dislocations, which impede dislocation movement consistency, causing a higher hardening rate at the origin of harmful blue brittleness of steels. Moreover, the hardening effect is continuous at temperatures 50~75 °C higher than the upper limit of DSA [1], at which our high-temperature (HT) fatigue tests were conducted.

The LCS investigated in this work is the main body material of coke drums. During the process of coke production, the temperature changes from RT to 400 °C. Components of coke drums undergo tensile–tensile cyclic loading during the production process. Dislocation motion is affected by the Cottrell atmosphere at this temperature [5]. Most importantly, fatigue strength and fatigue life are highly related to dislocation density, which makes it important to reveal how dislocation density evolves under cyclic loading at RT and HT. Under 400 °C, mechanical properties are influenced by the DSA effect, including brittleness, hardening, etc.; the mechanism causing these variations has not previously been fully understood, not to mention variations in the fatigue life. Most previous works demonstrated that the fatigue life of LCS decreases at elevated temperatures [6,7,8]. However, the results of this study indicate that the fatigue life increases at HT (400 °C) compared to RT. This phenomenon, which has not been widely reported, urgently necessitated an investigation into its underlying mechanism.

Two well-established techniques have been adopted to develop approaches for dislocation measurement on a mesoscopic scale, electron backscatter diffraction (EBSD) and high angular resolution EBSD (HR-EBSD) and X-ray diffraction (XRD). EBSD uses local orientations to compute the curvature of each pixel, after which the components of Nye’s tensor can be computed. HR-EBSD uses a digital image correlation (DIC) method to determine relative misorientation of patterns in EBSD as well as elastic strains, with an accuracy on the order of $1\times 10^{-4}$ [9,10,11]. The diffraction line profiles measured by XRD provide information about dislocation density [12]. Since the measured region of XRD is on a macroscale, the dislocation density computed is considered to be a microstructure-averaged value. It has been demonstrated that dislocation density can be evaluated by XRD not only for nanocrystalline grains, but also for larger grains such as tens of micrometers [13] based on a modified Williamson–Hall (MWH) and a modified Warren–Averbach (MWA) method [14,15,16,17,18].

A polycrystalline aggregate generates dislocations to fulfill plastic deformation, accompanied by annihilation and movement [19]. Heterogeneous deformation at microscopic-scale levels up the strain gradients across or inside grains is inherent to polycrystalline materials [20,21,22]. The dislocations that have a net non-zero Burgers vector can induce localized curvatures of crystal lattice, which are referred to as geometrically necessary dislocations (GNDs). In contrast, the dislocations that have a net zero Burgers vector and do not generate curvatures are referred to as statistically stored dislocations (SSDs). These two kinds of dislocations have a tendency to form lowest-energy configurations by dislocation walls, cells, and boundaries [23]. XRD measures the overall density of all kinds of dislocations, i.e., the sum of GNDs and SSDs, since stress fields induced by dislocation cores reflect on the diffraction line profile. However, EBSD/HR-EBSD measures the dislocation density in a different way, which is based on differentials of localized lattice orientations; thus only GNDs can be evaluated by EBSD/HR-EBSD, theoretically. Since orientation of each pixel is geometrically averaged, curvature is affected by size of the pixels. Therefore, to evaluate the variation of GND density via EBSD/HR-EBSD, the measurement parameters should maintain consistency for all specimens, including step size, exposure time, etc.

The dislocation density in a polycrystalline aggregate is related to stored energy, which has served as a criterion for fatigue failure in some works. Pangborn et al. [24] have demonstrated, using XRD techniques, that there is a critical value of excess dislocation density for 2024 aluminum alloy, $\rho^{*}$, beyond which fracture occurs. Furthermore, a critical value of dislocation density has been connected with the Huffman damage model; a good correlation can be achieved to predict the fatigue life of steels [25]. In many cases, GND density evaluated by EBSD/HR-EBSD has been used to represent variation of microstructure under plastic deformation. However there are limited investigation about change of GND density under cyclic loading. Combining EBSD/HR-EBSD with XRD, one can measure SSD and GND densities separately to reveal effects of total dislocation density, GND density, and SSD density on behaviors of metals [12]. 

In this work, we conducted RT and HT (400 °C) fatigue tests for LCS with a stress ratio of R=0. To facilitate clearer descriptions, HT will be used hereafter to refer to 400 °C. Three techniques were employed to investigate the variation in the dislocation density after a certain number of cycles. Using the XRD technique and the MWA method, the total dislocation density was determined, and the GND density was computed using EBSD/HR-EBSD techniques based on the principle of lowest energy. The variation in the dislocation density was furtherly analyzed to explain the mechanism of prolonged fatigue life at HT. As a supplement to this work, RT tensile tests followed by heat treatment at HT have been carried out to reveal dislocation annihilation activity.

## 2. Materials and Experiments

### 2.1. Material

**Figure 1:**
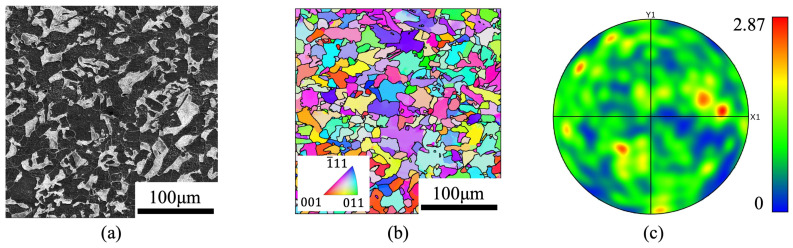
(**a**) Microstructure of the ferritic–pearlitic steel after polishing and etching, (**b**) EBSD results, and (**c**) the pole figure corresponding to (**b**).

The material investigated in this work is ferritic–pearlitic steel (0.2% C in wt%); detailed composition is listed in Table 1. The as-received material is extruded round bars with a diameter of 16 mm, which were fully annealed followed by natural aging to obtain equiaxed and texture-free ferrite and pearlite grains (for the sake of simplicity, we refer to regions with cementite lamella as ‘pearlite grains’), as shown in Figure 1a. The surface metallographic structure, consisting of 69.84% ferrite and 30.16% pearlite, is revealed by grinding, mechanical polishing and etching.

### 2.2. Experiments

Initial tensile tests were performed to characterize the mechanical behavior of the LCS at RT and HT, then fatigue tests were conducted under force control. Subsequently, X-ray diffraction (XRD) was utilized to measure diffraction line profiles for dislocation density evaluation. Finally, EBSD was conducted to determine the KAM and GND in specimens subjected to various conditions and cycle numbers. Afterwards, HR-EBSD was conducted to visualize the distribution of KAM and GND, as well as to compute the elastic strains.

#### 2.2.1. Tensile and Fatigue Tests

**Figure 2:**
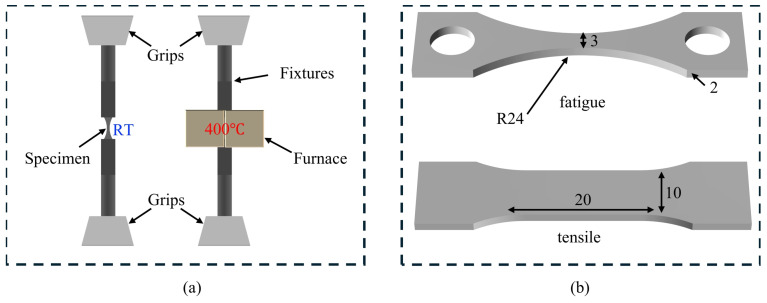
(**a**) The diagram of RT and HT fatigue tests; specimens were mounted on the fixtures. (**b**) The dimensions of the fatigue and tensile specimens.

LCS specimens for tensile and fatigue tests were cut from the round bars using an electric discharge machine with longitudinal axis parallel to the extruding direction. The dimensions of the tensile and the fatigue specimens were determined according to GB/T 26076–2010 standard [26] and GB/T 228.1-2021 standard [27], as shown in Figure 2b. RT and HT tensile/fatigue tests were conducted by a 100 KN Servohydraulic Test System (MTS, Eden Prairie, MN, USA). The fatigue tests were carried out with a sinusoidal signal at a frequency f = 20 HZ and a stress ratio of R = 0. The experimental conditions were chosen by the production process of coke drums, as detailed in Table 2. HT fatigue tests were achieved by MTS Model 653.2 High-Temperature Furnace (MTS, Eden Prairie, MN, USA), inside which the hot zone is 50 mm in height. The sample was mounted on a pair of fixtures as illustrated in Figure 2a, which can be mounted on MTS 647 Hydraulic Grips (MTS, Eden Prairie, MN, USA). Before HT fatigue tests, the sample was held in the furnace for 30 min until the temperature was 400 ± 1 °C. Tensile testing was conducted at a constant strain rate of 0.005/s. Another tensile test was carried out specifically to evaluate the effect of heat recovery under HT; thus the specimen was loaded to 400 MPa and unloaded, then heated at HT for 24 h. Dislocation densities of the fatigue and tensile specimens were measured by XRD and EBSD techniques; details will be discussed in the following sections.

#### 2.2.2. XRD Tests

Five fatigue specimens were measured by XRD, including the as-received specimen, the specimen subjected to HT fatigue test for 200,000 cycles, the specimen subjected to HT fatigue test for 5,000,000 cycles, the specimen subjected to HT fatigue test for 10,000,000 cycles, and the specimen subjected to RT fatigue test for 180,265 cycles (until failure). Specimen preparation procedure was grinding followed by mechanical polishing and electropolishing to remove the surface residual stress; the same procedure was used for EBSD specimens. To ensure that surface defects did not affect the experimental results, all surfaces of the sample were treated using the aforementioned procedures, with the upper surface designated as the testing surface.

A High power 9 KW X-ray diffractometer (Rigaku, Tokyo, Japan) with Cukα radiation was used for XRD tests. To cover the six peaks of (110), (200), (211), (220), (310) and (222), the diffraction lines were collected from 35° to 145° of 2θ with a step size of 0.002° [13]. The diffraction profiles were firstly fitted by pseudo-Voigt function, then corrected by splitting Κα2 and Kα1. The line profile of standard LaB6 powder sample was collected with the same setting. The classical Stokes method [28] was written to an in-house code to deconvolute the LaB6 profile from samples to eliminate the instrument broadening effect. 

Experiments have shown that dislocation density can be manifested by strain broadening, which causes distortion of XRD profiles. The XRD profile, described by a special logarithmic series expansion of the Fourier coefficients, is related to dislocation density [18]. Please refer to the Appendix A. for the detailed fitting process.

Transformation of Equation (A9) gives:

(1) $$X\left(L\right)/L^{2}=\left(pпb^{2}/2\right)\left(\ln R_{e}-\ln L\right)$$

The dislocation density can thus be determined by linear fitting $X\left(L\right)/L^{2}$ against lnL. 

#### 2.2.3. EBSD Tests

EBSD tests were performed using a JEOL IT800-SHL field-emission SEM (Tokyo, Japan) equipped with an Oxford Symmetry S2 detector. The step size was 0.3 μm, and the region was 300 × 300 μm^2^, with each region containing 1,000,000 pixels. The pattern was clear enough to calculate the orientations with an exposure time of 0.5 ms, and the collection time was 500 s for each region. The Euler angles, KAM values and GND values [29] were computed using MTEX [30], a free MATLAB toolbox for analyzing and modeling crystallographic textures using EBSD or pole figure data.

In common EBSD results, the elastic strain tensor is neglected, which means five of the components are accessible: $\alpha_{12}$, $\alpha_{13}$, $\alpha_{21}$, $\alpha_{23}$ and $\alpha_{33}$.

Considering 48 independent slip systems in BCC structure, there are 4 pure screw type and 48 pure edge type dislocations to be considered. Therefore, it is impossible to determine the dislocation density of each slip system. A lowest-energy principle is adopted in this work to estimate the GND density based on the five known components of Nye’s tensor, and the energy of screw and edge energy is estimated by:

(2) $$U_{\mathrm{screw}}=\frac{Gb^{2}}{4\pi }\ln\frac{R}{r_{0}},\;U_{screw}=\left(1-\upsilon \right)U_{\mathrm{edge}}$$

where *G* is the shear modules, *b* is the Burgers vector, *R* is the core radius of the dislocation, and υ is the Possion’s ratio. It is convenient to assume $U_{\mathrm{edge}}=1$ and $U_{\mathrm{screw}}=0.7$. The lowest-energy principle is expressed by Equation (3):

(3) $$\sum_{t=1}^{N}u^{t}\rho^{t}=min$$

where *N* is the number of the slip system. 

Wilkinson et al. [11] described that increasing the separation size (Δx) elevates the probability of crossing multiple GNBs, resulting in a lower estimation of lattice curvature. The GND density computed using the EBSD technique yields relative values depending on Δx, which means the separation size needs to be constant when comparing GND values in different specimens.

#### 2.2.4. HR-EBSD Tests

HR-EBSD tests were conducted using the same equipment as the EBSD tests. Since the sample surface was electropolished to remove stress, the quality of diffraction patterns met the requirements for HR-EBSD computation. The collected patterns were 16-bit depth with a resolution of 1028×1028 pixels. To obtain a finer analysis of GND distribution, a step size of 0.2 μm was used to collect a 75 × 75 μm^2^ region for each specimen. HR-EBSD computation was performed using the ATEX software (Version 4.12) [31], which is an analysis tool for electron and XRD developed by Jean-Jacques Fundenberger and Benoît Beausir.

The disorientation and the elastic strain tensor can be determined using digital correlation method (DIC). Long exposure time (30 times more than the common EBSD tests) and good surface quality ensure the accuracy of HR-EBSD tests, as detailed by Ernould et al. [9]. The detailed definitions of KAM and the DIC method are provided in Appendix B. According to Equation (A14), $\alpha_{13}$, $\alpha_{23}$ and $\alpha_{33}$ can be computed including the elastic strain, while the other two components ($\alpha_{12}$ and $\alpha_{21}$) hold the same requirements as common EBSD. After DIC calculation for every pattern, the orientation of pixels can be re-indexed to compute GND density using the lowest-energy principle using an in-house MATLAB code. The results will be presented and discussed in the following sections.

## 3. Results

### 3.1. Fatigue and Tensile Tests

**Figure 3:**
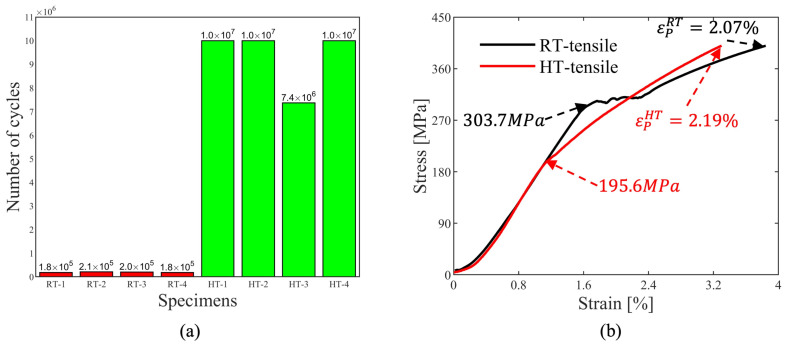
(**a**) Results of fatigue tests at RT and HT, and (**b**) stress–strain curves of tensile tests.

Under R = 0 cyclic loading, LCS exhibits cyclic softening characteristics at both RT and HT with different softening rates. More importantly, fatigue life in RT and HT tests is quite different. As shown in Figure 3a, the mean fatigue life of the four RT specimens was 190,873 cycles, with individual percentage deviations of 5.56%, 7.92%, 5.44% and 7.79%. However, at HT three specimens reached 10,000,000 cycles without any fatigue cracks, with only one specimen fractured after 7,365,846 cycles. This substantial enhancement of fatigue life from RT to HT has not been widely reported. 

The tensile stress–strain curves at RT and HT until 400 MPa are shown in Figure 3b. The classic upper and lower yield points were observed during the RT tensile tests, attributed to interstitial atoms impeding dislocation movements resulting in the appearance of Lüders bands [32]. A well-known effect is the so-called DSA, originating from interstitial atoms, mostly carbon atoms in LCS [33]. In low-temperature regions, when dislocations interacted with fixed interstitial atoms, higher stress was required to move the pinned dislocations. After the dislocations overcame the fixed interstitial atoms, a lower stress was sufficient to drive the mobile dislocations, as evidenced by the RT tensile stress–strain curve shown in Figure 3b. At intermediate-temperature regions, the interstitial atoms had higher diffusion rates, sufficient enough to migrate to the nearest dislocation core and impede further movement of the dislocations, hardening the material in a dynamic way, leaving jerky flow and serrated flow curves. At higher-temperature regions, the interstitial atoms possessed enough energy to move along the dislocations; no apparent serration was observed in the tension curves. The yield stress decreased with the increased temperature, and the ductility increased. However, somewhere between the intermediate- and the high-temperature regions, there existed a small gap where the serration vanished, yet the ultimate tensile strength stayed at a high level. As observed by Dolzhenkov [1], the brittleness/hardening effect continues at 50–75 °C higher than the temperature where the serrations disappear. As illustrated in Figure 3b, no serration was observed in the tensile curve at HT, and the hardening rate was higher than at RT, indicating that 400 °C marked the transition from the intermediate- to the high-temperature regions of the LCS specimens.

The fatigue test results under 0–400 MPa indicated a significant improvement in the fatigue life at HT. This phenomenon is likely related to dislocation movements. Moreover, HT environments can elevate the rate of dislocation annihilation, with cyclic deformation also playing a role. In the following sections, the dislocation density measured by XRD, EBSD and HR-EBSD will be presented. 

### 3.2. Total Dislocation-Density Values under Different Experimental Conditions

The strain field of a dislocation core decays with $r^{-1},$ as it is a long-range effect, whereas interstitial atoms exhibit short-range effects that decay with $r^{-3}$ [34]. This means that the primary lattice distortions captured by XRD are induced by dislocations, which are a combination of GNDs and SSDs.

**Figure 4:**
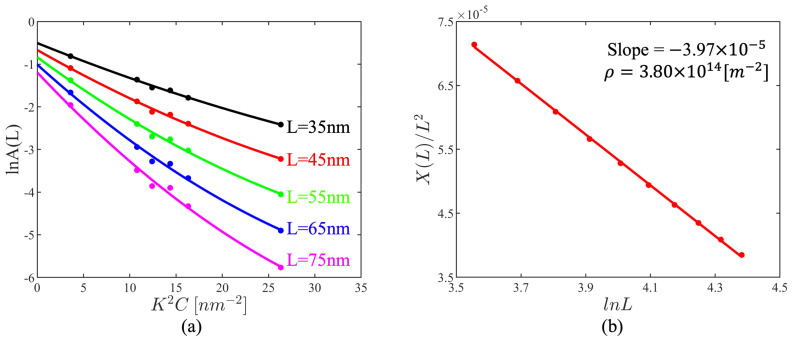
(**a**) Modified Warren–Averbach (MWA) plot of Equation (A8) of the HT fatigued specimen after 200,000 cycles. (**b**) Linear fitting results of Equation (A8) of the HT fatigued specimen after 200,000 cycles; the total dislocation density can be derived from the slope.

A self-written MATLAB code was used to eliminate any instrumental broadening effects and to perform the aforementioned calculations. The real part of the Fourier coefficients A(L) can be computed from the line profile, after which the X(L) value can be derived by fitting with Equation (A8), as illustrated in Figure 4a. Subsequently, the dislocation density can be computed by linear fitting with Equation (1), as shown in Figure 4b.

**Figure 5:**
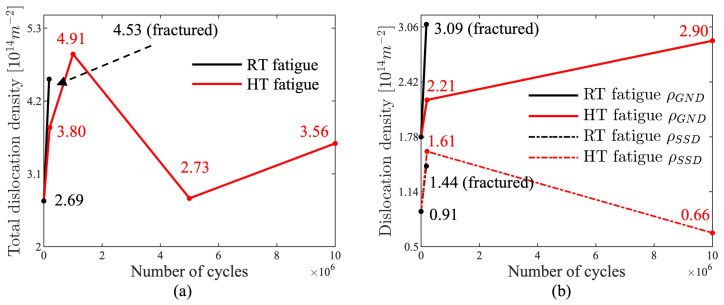
(**a**) Total dislocation-density variation of RT and HT fatigue specimens at different cyclic loading counts. (**b**) GND- and SSD-density variations of RT and HT fatigue specimens at different cyclic loading counts.

**Figure 6:**
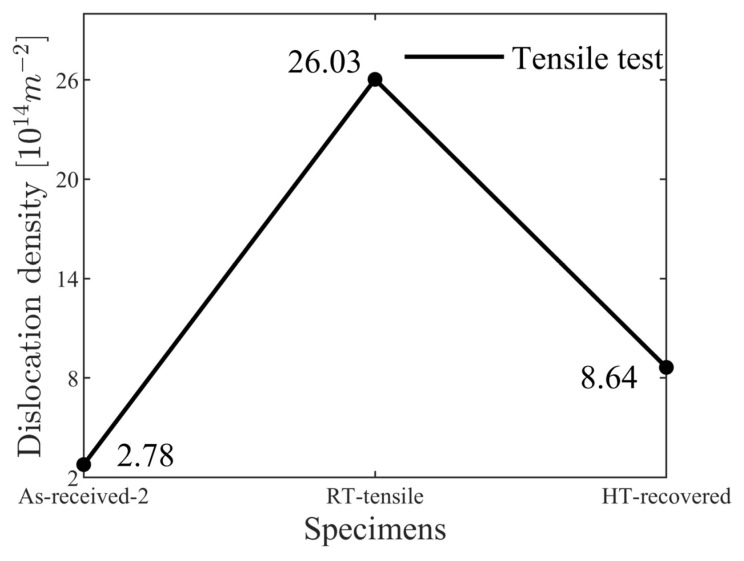
Dislocation-density variation of RT tensile specimen as-received, after tensile stress, and following HT recovery.

The variations of the dislocation densities in the fatigue and tensile tests are shown in Figure 5a and Figure 6. The dislocation densities of the two as-received specimens were $2.69\times 10^{14}\;m^{-2}$ and $2.78\times 10^{14}\;m^{-2}$ with an error of 3.3%, validating the accuracy of the dislocation density evaluated using XRD with the in-house code. The RT specimen in the fatigue test fractured after 180,265 cyclic loadings at 0~400 MPa, with its dislocation density increased to $4.53\times 10^{14}\;m^{-2}$. 

The variations of the dislocation densities at HT are more complex. As shown in Figure 5a, the dislocation density initially increased to $3.8\times 10^{14}\;m^{-2}$ after 200,000 cycles, which is less than in the RT tests. The dislocation density continued to increase until 1,000,000 cycles, to a value of $4.91\times 10^{14}\;m^{-2}$, which is greater than that of the fractured fatigue specimen at RT. After 5,000,000 cycles, the dislocation density had decreased to a value between those of the two as-received specimens, indicating a strong recovery effect to decrease the dislocation density. Finally, after 10,000,000 cycles, the dislocation density had increased at a slower rate compared to the rate measured during the first 200,000 cycles of the fatigue test. Overall, during the HT fatigue test the dislocation density exhibited a fluctuating trend, with periods of increase and decrease, ultimately measuring $3.56\times 10^{14}\;m^{-2}$ after 10,000,000 cycles. 

The quasi-in situ tensile tests conducted at RT with HT recovery show the recovery rate of dislocations, as shown in Figure 6. The as-received specimen had an initial dislocation density of $2.78\times 10^{14}\;m^{-2}$, which is approximately equal to that of the quasi-in situ HT fatigue specimen. After applying a 400 MPa tensile stress, the dislocation density had increased to $26.03\times 10^{14}\;m^{-2}$, which is 9.36 times the initial value before the tensile test. After recovery at 400 °C for 24 h, the dislocation density had decreased to $8.64\times 10^{14}\;m^{-2}$, which is 3.11 times the initial state. The total dislocation density recovered by $17.39\times 10^{14}\;m^{-2}$ after 24 h at 400 °C without external force, indicating the presence of climb-controlled recovery during HT fatigue tests. However, compared to the RT fatigue test, which lasted 2.5 h for 180,265 cycles, the dislocation density decreased by $21.50\times 10^{14}$ m^−2^, showing that the annihilation rate was much faster than heat recovery. Therefore, despite the presence of heat recovery, the dominant mechanism of dislocation annihilation was cyclic loading.

### 3.3. Evolution of GND Density during Fatigue Tests 

The GND density was evaluated using EBSD tests to measure the specimen surfaces after different cyclic loadings under both RT and HT. The EBSD results were analyzed using MTEX [30], with the KAM and the GND density computed based on Equations (2) and (3). The step size and exposure time remained constant to ensure that the KAM and GND values were comparable between the specimens. 

**Figure 7:**
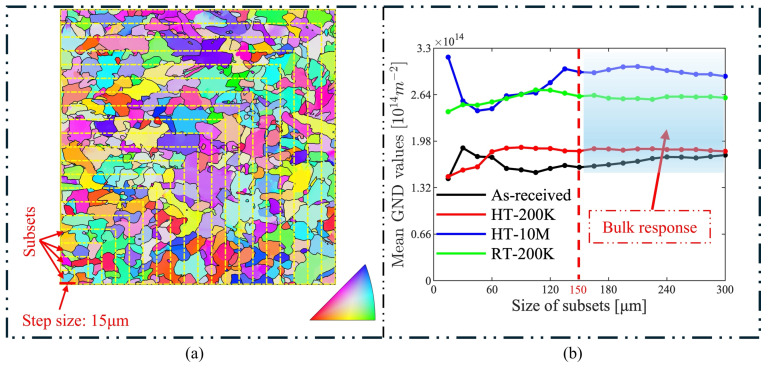
(**a**) The illustration of the 20 subsets; the step size is 15 μm from left bottom corner. (**b**) The mean GND-value response of the subsets of the fatigue specimens.

Before analyzing the EBSD results, steps were taken to ensure that the mean KAM/GND values were truly representative of the bulk response of the material by validating the statistical significance of the results. A self-written MATLAB code was used to create subsets from the current EBSD data and to compute the GND values for each subset. For this validation work, the EBSD data covered an area of 300 × 300 μm^2^, and the subsets were created from the bottom left corner with a step size of 15 μm as illustrated in Figure 7a, creating 20 subsets for the EBSD data. The KAM/GND values were computed for every subset to validate if the area was sufficient to represent the bulk response. An example is shown in Figure 7b, where the mean GND values for each subset in the four fatigue specimens were plotted. From Figure 7, it can be observed that when the subset size was below 150 µm, the GND values were highly unstable and varied with the subset size. This indicates that the region was too small to capture the overall response of the material, and the measured values lacked generality. When the subset size exceeded 150 µm, the GND values of the four samples tended to stabilize. From the results, we can infer that the selected regions of the EBSD results were large enough to represent the bulk response of the specimens.

**Figure 8:**
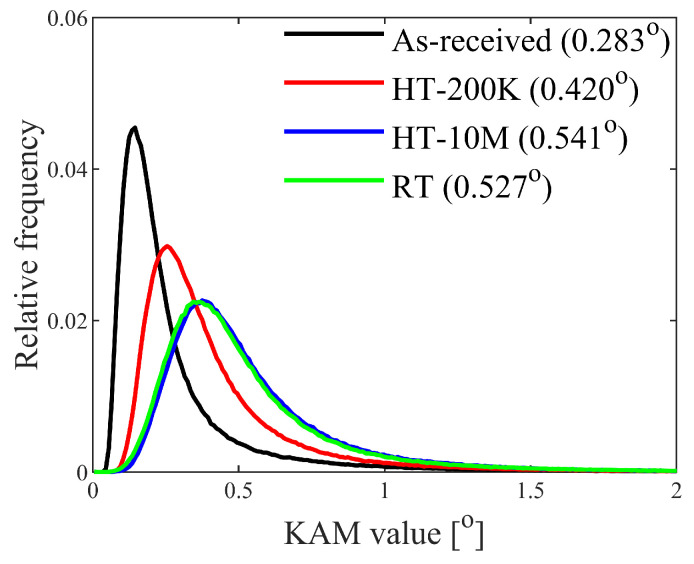
The KAM relative-frequency distribution of the four fatigue specimens.

The as-received specimens were annealed to a relative lower average KAM ($KAM_{ave}$) value of 0.283, as shown in Figure 8. After being fatigued for 200,000 cycles under HT, the $KAM_{ave}$ had increased to 0.420. The value continued to increase with cyclic loading, reaching 0.541 after 10,000,000 cycles. After the RT fatigue tests, the $KAM_{ave}$ value had increased from 0.283 to 0.527, which is only 2.59% less than the HT specimen after 10,000,000 cycles. 

**Figure 9:**
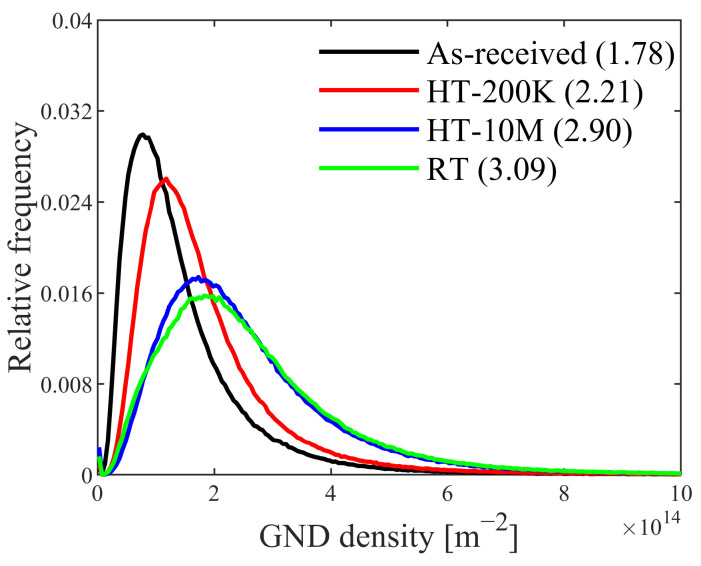
The GND relative-frequency distribution of the four fatigue specimens.

The average GND density ($\rho_{GND}$) of the as-received specimen was $1.78\times 10^{14}\;m^{-2}$, as shown in Figure 9. After 200,000 cyclic loadings under HT, the $\rho_{GND}$ had increased to $2.21\times 10^{14}\;m^{-2}$, which is 1.24 times the original value. The final GND density was $2.90\times 10^{14}\;m^{-2}$, which is 1.63 times the original. After 180,265 cyclic loadings under RT, the $\rho_{GND}$ had increased to $3.09\times 10^{14}\;m^{-2}$, which is higher than the final value of the HT fatigued specimen. Although the general trends of KAM and GND evolution are similar, the detailed increments differ. Moreover, the mean GND values computed by EBSD are lower than the dislocation values determined from XRD, which can be attributed to several factors: SSD cannot be measured by EBSD, the lowest-energy principle assumes that SSD are in the lowest density configuration [23], and the step size of EBSD tests can affect the GND value.

**Figure 10:**
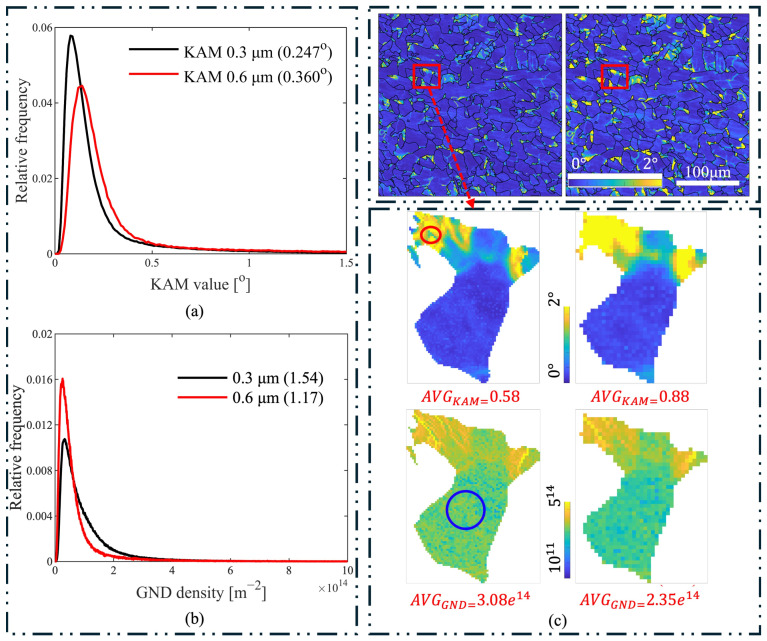
(**a**) The KAM relative-frequency distribution of the as-received specimen with step sizes of 0.3 and 0.6 μm; (**b**) the GND density relative-frequency distribution of the as-received specimen with step sizes of 0.3 and 0.6 μm; (**c**) KAM and GND distributions of a single grain measured with step sizes of 0.3 and 0.6 μm.

Dislocation dipoles, planar dislocation loops, and other self-terminating dislocation structures fully contained within reference areas make no net contribution to Nye’s tensor, hence the dislocation density inside the selected areas could not be measured by EBSD [23]. To demonstrate the effect of step size, another as-received specimen was measured with 0.3 μm and 0.6 μm step sizes for the same region, and the relative-frequency distributions of the KAM and GND values are plotted in Figure 10a,b. The variation of GND with step size is consistent with the assumption [12] that GND decreases with increasing step size. However, the KAM values varied differently. One grain in the map was chosen to visualize the influence of step size on the KAM and GND values. As shown in Figure 10c, more detailed distributions of KAM and GND can be visualized on the 0.3 μm step-size map compared to the 0.6 μm. In KAM distribution maps, the value is evaluated based on the averaged disorientation values of neighboring pixels within a certain range, meaning KAM value is lower only if a large portion of neighboring pixels have similar orientations. Consequently, small areas of low KAM values (as marked with a red circle in Figure 10c) cannot be detected with larger step sizes, causing overall KAM values to increase with step size. In contrast, GND values are computed based on the curvature tensor defined in Equation (A11), which correlates with individual pixels, allowing detailed disorientations to be revealed with smaller step sizes, as shown by the blue circle in Figure 10c. 

### 3.4. Detailed KAM and GND Distributions Computed from Re-Indexed HR-EBSD Results

**Figure 11:**
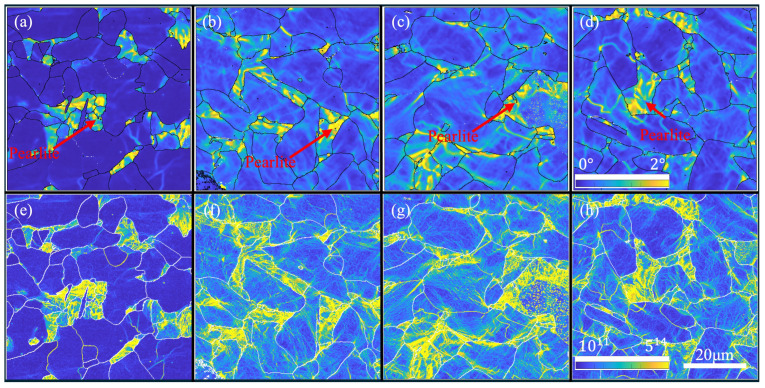
KAM and GND distribution maps of the re-indexed HR-EBSD results, calculated by ATEX and MTEX using the lowest dislocation energy principle; the KAM values are evaluated by the order of three. Maps (**a**–**d**) are the KAM values for specimens, including the as-received specimen, the specimen subjected to HT fatigue test for 200,000 cycles, the specimen subjected to HT fatigue test for 10,000,000 cycles and the specimen subjected to RT fatigue test for 200,000 cycles, respectively, and (**e**–**h**) are the GDN values of the four specimens in the same order.

HR-EBSD tests were conducted on the four fatigue specimens to reveal the detailed distributions of KAM and GND. A re-indexing procedure was performed using ATEX software [31], and the generated EBSD files were used to compute the KAM and GND distributions using the same methods as in Section 3.3. The results are shown in Figure 11.

It becomes clear from Figure 11 that KAM and GND were at high levels in the pearlite regions of the undeformed specimen and remained nearly unchanged throughout the fatigue tests. In the ferrite grains, KAM and GND were lowest, with no distinctive GND substructure observed. With increasing cycles, both KAM and GND increased, indicating that lattice curvature had been promoted to maintain strain compatibility across variously oriented grains [19]. The accumulation of KAM and GND followed the same trend. It is further observed that KAM and GND in the ferrite grains were more uniformly distributed in the HT fatigue specimens, as illustrated in Figure 11b,c,f,g. In contrast, some grains in the RT specimens remained at relatively low KAM and GND values, showing a higher degree of non-uniformity compared to HT specimens, as illustrated between Figure 11b–d,f–h, even though the two specimens underwent a similar number of cycles.

**Figure 12:**
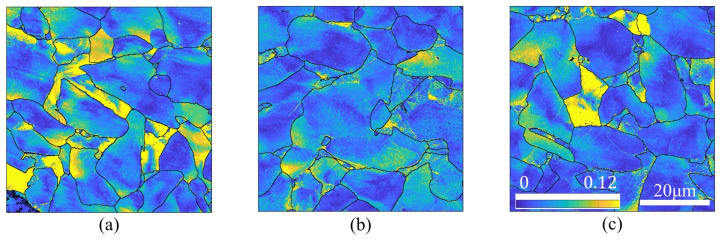
Von Mises equivalent strain distribution maps of the specimens: (**a**) subjected to HT fatigue test for 200,000 cycles, (**b**) subjected to HT fatigue test for 10,000,000 cycles, and (**c**) subjected to RT fatigue test for 200,000 cycles.

Furthermore, elastic strains were computed based on the distortion of the EBSD data patterns using ATEX. As shown in Figure 12, the distribution of von Mises equivalent strain in the RT and HT fatigued specimens is similar to KAM and GND, with most strain concentrated near triple junctions or adjacent to grain boundaries. 

## 4. Discussion

### 4.1. Dislocation Motion Mechanism

Variation in dislocation density during cyclic loading can be subdivided into three stages [35,36]: multiplication stage, stabilization stage, and final rupture stage. During the multiplication stage, the dislocation density increases rapidly as dislocation entanglement serves as an obstacle, hardening the material. After that, the dislocation density in some metals increases at a constant rate during the stabilization stage, while in other materials the multiplication and annihilation rates of the dislocations balance each other, depending on loading conditions and material properties [24,35]. Defects dislocate consistently during this stage, leading to damage accumulation manifested by extrusions and intrusions on the sample surface, contributing to reduction of the dislocation density. Additionally, two other mechanisms can decrease the dislocation density: (a) annihilation of the dislocations and (b) entrapment of the dislocations in boundaries, such as subgrain boundaries, etc. [37]. Under most circumstances, the reduction of the dislocations by these mechanisms can be measured by XRD. From the XRD results, we see that the dislocation density in the HT fatigue tests had reached dynamic equilibrium after the first 200,000 cycles, indicating similar multiplication and annihilation rates. External stress is the main driving force for dislocation motion in both RT and HT fatigue tests. Caillard [4] observed pure climb-controlled recovery of prismatic loops in FeCrC at over 500 °C, but not at 400 °C in low-carbon steels. 

Since total dislocation density and GND density can be measured by XRD and EBSD, SSD density can be computed based on the two measurements. The results shown in Figure 5b indicate that the GND density increased during the stabilization stage, while the SSD density decreased. It should be noted that GND values are related to step size. Unlike the trend observed in KAM concerning step size, GND increases as step size decreases. Therefore, the measured GND data represents relative values rather than absolute ones, as shown in Figure 10. This implies that further reducing the step size might yield higher GND values, resulting in lower SSD values. Nonetheless, the overall trend of GND and SSD with respect to cyclic loading is valid.

Plastic deformation of LCS is controlled by motion of the screw dislocations due to their complex mechanisms and immobility. Based on the XRD results, the fraction of edge dislocations decreased during the HT fatigue test, from a maximum value of 0.75 to 0.55, indicating that the annihilation rate of the edge dislocations was higher than that of the screw dislocations, meaning the motion of the screw dislocations was impeded. For other BCC metals, screw dislocations and cross-slip are promoted at elevated temperatures, allowing dislocation dipoles to effectively annihilate, decreasing screw dislocation density. Therefore, in LCS, the mobility of the screw dislocations is lower than in other BCC materials, such as high-entropy alloys and titanium alloys [38]. Impeding of screw dislocations results in continuous hardening of LCS at HT.

### 4.2. Cyclic Response of LCS Specimens under RT and HT Cyclic Loading

As shown in Figure 3b, the yield stress at RT was 303.7 MPa, which decreased to 195.6 MPa at HT. The plastic strains during the tensile tests were 2.07% and 2.19%, respectively, indicating higher plastic deformation at the beginning of the fatigue tests for the HT specimens compared to the RT specimens. Given that the stress range of the fatigue tests was 0–400 MPa, the plastic deformation during the fatigue tests was manifested by the increment of the maximum displacement value of the current cycle, $S^{n}$, where *n* is the number of the current cycle. 

**Figure 13:**
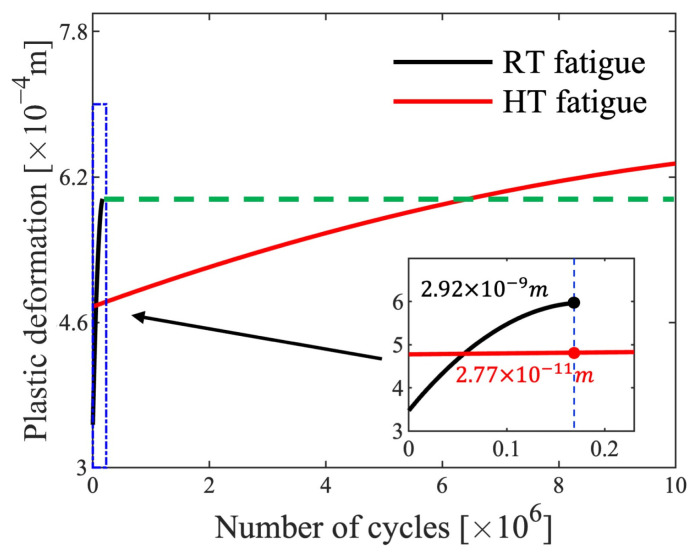
The maximum plastic deformation displacement of each cycle.

The plastic deformation of the RT and HT samples over each cycle is shown in Figure 13. At the beginning point, the deformation at HT was higher than that at RT. By the end of the stabilization stage of the RT fatigue tests, the increment rate of the plastic deformation was ${2.92\times 10}^{-9}\;m/cycle$, while it was ${2.77\times 10}^{-11}\;m/cycle$ at the same number of cycles for the HT fatigue tests. The initial stage of the HT fatigue tests contributed 72.28% of the total plastic deformation during the tests due to the lowered yield stress at HT, whereas the RT value was 55.90%.

**Figure 14:**
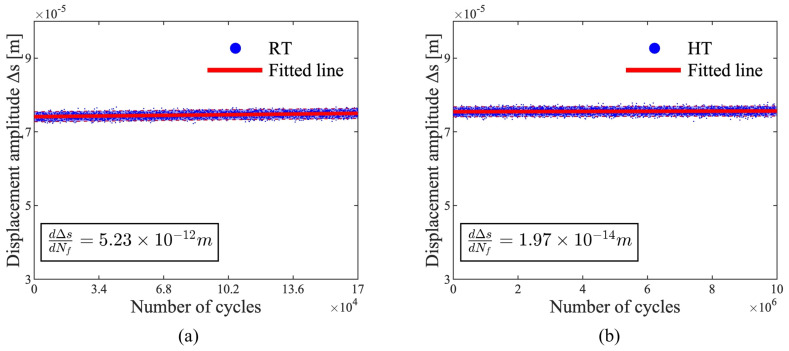
(**a**) Evolution of displacement amplitude of RT fatigue test, and (**b**) evolution of displacement amplitude of HT fatigue test with confidence interval set at 95% (shaded area).

Furthermore, the evolution of displacement amplitude, ∆S is shown in Figure 14, which can be used to determine cyclic softening/hardening characteristics. ∆S increased with increasing cycles for both the RT and HT fatigue specimens, indicating cyclic softening, yet the rates varied significantly, at ${5.23\times 10}^{-12}\;$ m/cycle and ${1.97\times 10}^{-14}\;$ m/cycle, respectively. This suggests that the plastic deformation mechanism changed at HT for the LCS specimens, evidenced by the significant decrease of the softening rate during the cyclic loading compared with RT.

As previously discussed, the dislocation motion mechanism changes with elevated temperature. Above the DSA upper-limit temperature range, screw dislocations move following the ‘high-temperature Peierls mechanism,’ similar to low-temperature motion observed in ferrite. At 400 °C, rapid dislocation gliding avalanches are not observed in smooth tensile curves, implying that the steady motion rate of the screw dislocations under the ‘high-temperature Peierls mechanism’ is sufficient to accommodate imposed deformation. However, screw dislocation mobility remains impeded at 400 °C, leading to a higher hardening rate in tensile tests and an increased fraction of screw dislocations under HT cyclic loading. 

### 4.3. GND, KAM and Plastic Deformation

EBSD and HR-EBSD results provide information on microstructure variations in terms of lattice orientations and elastic strains. This work demonstrates that the GND values can represent the bulk properties of the material within a 150 × 150 μm^2^ area.

**Figure 15:**
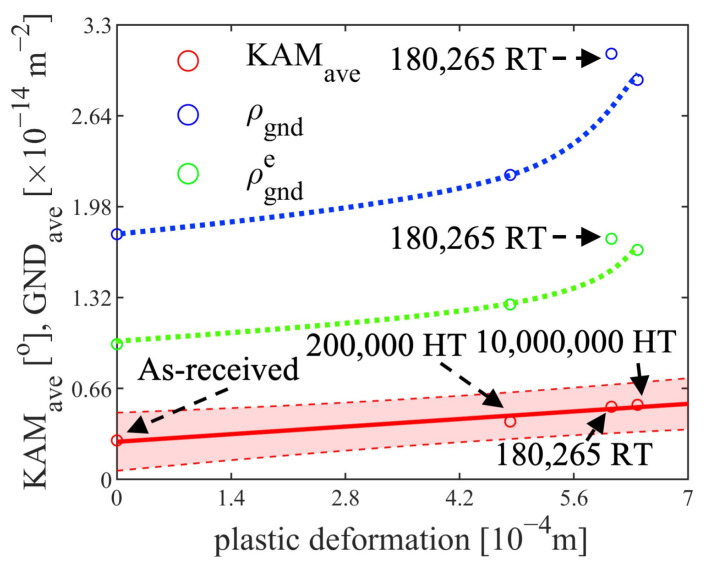
The relationship between $KAM_{ave}$, $\rho_{GND}$, $\rho_{GND}^{e}$ and plastic deformation of the four fatigue specimens, with confidence interval set at 95% (shaded area).

A linear relationship between the plastic deformation and the averaged KAM value was observed from the EBSD results. As shown in Figure 8 and Figure 14, the ${KAM}_{ave}$ of the 200,000-cycle HT specimen was 0.420, corresponding to a plastic deformation of $4.82\times 10^{-4}\;m$. The ${KAM}_{ave}$ of the 180,265-cycle RT specimen was 0.527 with a plastic deformation of $6.06\times 10^{-4}\;m$, and the ${KAM}_{ave}$ of the 10,000,000-cycle HT specimen was 0.541 with a plastic deformation of $6.38\times 10^{-4}\;m$. The relationships are plotted in Figure 15. However, the $\rho_{GND}$ did not follow a linear relationship with plastic deformation. By assuming two non-orthogonal activated slip systems carrying plastic deformation, $KAM_{ave}$ can be written as:

(4) $$KAM_{ave}=\frac{1}{S_{\mathrm{Grain}}}\int_{0}^{2\pi }{bKAM\left(\varphi \right)\frac{1}{2}\left(\frac{D_{\mathrm{Grain}}}{2}\right)}^{2}d\varphi =\frac{a\zeta \varepsilon^{p}}{2\pi D_{\mathrm{Grain}}}\int_{0}^{2\pi }\left(\left|\cos\varphi \right|+\left|\sin\varphi \right|\right)d\varphi =\frac{4a\zeta \varepsilon^{p}}{\pi D_{\mathrm{Grain}}}$$

where $\zeta \left(\varphi_{1},\varphi_{2}\right)=\left|\sin\left(\varphi_{1}+\varphi_{2}\right)/\cos\left(\varphi_{1}-\varphi_{2}\right)\right|$ is the factor, including the azimuthal angles $\varphi_{1}$ and $\varphi_{2}$ of the slip systems [39]. The ${KAM}_{ave}$ is proportional to the nominal plastic strains, which can be further written as:

(5) $$KAM_{ave}=\frac{2ab}{\pi }\rho_{GND}^{e}$$

thus:

(6) $$\rho_{GND}^{e}=\frac{2\zeta \varepsilon^{p}}{bD}$$

where $\rho_{GND}^{e}$ is the edge GND density, a is the step size and *b* is the Burgers vector. Equations (4)–(6) imply that the $KAM_{ave}$ and the $\rho_{GND}^{e}$ are proportional to the plastic deformation. As shown in Figure 15, the combined ${KAM}_{ave}$ of the RT and HT fatigue specimens was proportional to the plastic deformation, while the $\rho_{\mathrm{GND}}^{e}$ was not. This discrepancy arises because $\rho_{GND}$ is fitted based on the lowest-energy principle, leading to higher-fitted results for screw dislocations, which may misestimate the amount of edge GND. Moreover, unlike the assumption of the two non-orthogonal slip systems during the plastic deformation, the GND fitting procedure assumes all 48 slip systems are activated, which aligns with realistic conditions. In contrast, the KAM values are computed based on localized orientations without fitting procedures, preserving a linear relationship.

HR-EBSD results are more accurate but occupy a huge amount of computation resources and disk space; thus HR-EBSD is more suitable for revealing localized information rather than bulk response. Comparing Figure 12b,c, finely distributed elastic strains inside the ferrite grains were observed in the HT fatigued specimen after 10,000,000 cycles, whereas the strains concentrated near the grain boundaries in the RT tests. Comparing Figure 12a,c, the distributions show substantial similarity, which means the elastic strain and the stress were relaxed during the HT fatigue tests, prolonging the fatigue life.

### 4.4. The Mechanism of the Enhancement of Fatigue Life

Based on the fatigue results, a substantial increase in fatigue strength was observed for the ferrite–pearlite LCS at HT, a phenomenon rarely reported. Unlike fatigue properties, tensile hardening of LCS under RT and HT was observed last century [1]. The prolonged fatigue life at HT reported here can be explained from a dislocation perspective. 

In the first stage of the fatigue tests, the plastic deformation in HT was higher than in RT, caused by the lower yield stress as indicated in Figure 3b and Figure 13. In the stabilization stage, under the influence of the ‘high-temperature Peierls mechanism’, the plastic deformation per cycle in HT was significantly lower than that in RT. Since the fatigue tests were conducted under the stress ratio R=0, the plastic deformation in each cycle can be treated as ratcheting deformation, defined as:

(7) $$S_{r}=\left(S_{L}^{P}+S_{R}^{P}\right)/2$$

where $S_{L}^{P}$ and $S_{R}^{P}$ are the maximum and minimum displacement during each cyclic loading. As inferred from Figure 13, the ratcheting deformation rates $\dot{S_{r}}$ decreased with increasing cycles but were not saturated in the RT and HT tests. The main difference between the two temperatures was the rate of the deformation. At HT, the lower rate resulted in less dislocation multiplication per cycle; localized plastic deformation was generated to fulfill the geometrical integrity. Under the influences of high temperature and the ‘high-temperature Peierls mechanism’, frequent cross slipping was activated in response to the deformation. Concurrently, the dislocation dipoles were annihilated due to thermal activation, increasing the screw dislocation fraction at HT, as observed from the XRD results. Since the line energy of screw dislocations is lower than that of edge dislocations (Equation (2)), the energy was lower during the HT fatigue tests, opposite to the higher edge fraction in the RT tests with higher energy. Therefore, the accumulated plastic deformation could not be used to represent the damage during cyclic loading because the plastic deformation and the $KAM_{ave}$ had changed with the temperatures.. 

However, the $\rho_{GND}$ and $\rho_{GND}^{e}$ shown in Figure 15 indicate that the RT specimen had higher plastic deformation compared with the HT specimens. The deformation coordination at high temperatures was improved, with increased cross-slip facilitating better deformation compatibility while maintaining the lowest possible GND to accommodate the strain. It is more useful to measure GND density to evaluate critical damage for fatigue initiation compared with other factors, such as total dislocation density, $KAM_{ave}$, elastic strain and accumulated plastic deformation. However, precise representation of fatigue damage based on GND still requires further research. 

Under the influence of carbon atoms, dislocation bursts and avalanches can cause slip traces on sample surfaces in a very short time, corresponding to serrations and jerky flow. Slip traces can further develop into extrusions and intrusions on the surfaces until fatigue crack initiates. At 400 °C, a smooth tensile curve appears, which means dislocations move in a viscous manner without the appearance of avalanches, resulting in less prominent slip traces, protecting the material from initiating cracks and prolonging the fatigue life.

## 5. Conclusions

Based on the above discussions, the following conclusions can be drawn:

1. The dominant reason for the dislocation annihilation during the high temperature fatigue tests was the external cyclic loading, not the heat recovery, as the heat recovery rate was significantly lower than the cyclic loading, which was proven by the quasi-in situ XRD tensile tests.
2. The fraction of screw dislocations increased during the HT fatigue tests with the help of ‘high-temperature Peierls mechanism’, forming the lower energy configuration.
3. The increment of the plastic deformation, i.e., the ratcheting rates of the HT fatigue tests in the stabilization stage, was significantly lower than in RT, and was a reason for the prolonged fatigue life at HT.
4. The smooth tensile curves under HT indicated no apparent bursts or avalanches, resulting in the less prominent slip traces, extrusions and intrusions and preserving fatigue crack from initiation and was another reason for the prolonged fatigue life at HT.
5. This newly discovered phenomenon of enhanced fatigue life at 400 °C can provide new insights for future designs of low-carbon steel components subjected to tension–tension cyclic loading, such as the main bodies of coke drums.

## Figures and Tables

**Table 1:** Chemical composition in wt%.

C	S	Si	P	Cr	Mn	Fe
0.2	0.22	0.392	0.03	0.038	0.547	Balance

**Table 2:** Experiment conditions.

Experiment	Wave Shape	Frequency (HZ)	Temperature (°C)	Stress (MPa)	Strain Rate (s^−1^)
RT fatigue	Sine	20	20	0~400	/
HT fatigue	Sine	20	400	0~400	/
Tensile	Monotonic	/	20	0~	0.005

## Data Availability

Data will be made available on request.

## Appendix A

Based on MWH method, the full width at half maximum (FWMH) of the diffraction peak profiles can be written as the sum of the two broadening effects [16]:

(A1) $$∆K=\frac{0.9}{D}+{\Delta K}^{D}$$

where ∆K=2cosθ(Δθ)/λ and θ, Δθ and λ are the diffraction angle, half of the FWHM of the diffraction peak and the wavelength of X-rays respectively. D is the average grain size or particle size, and ${\Delta K}^{D}$ is the strain contribution to peak broadening. By considering the dislocation density and the flux of it, Equation (A1) can be rewritten as:

(A2) $$\Delta K=\frac{0.9}{D}+{\left(\frac{\pi Ab^{2}}{2}\right)}^{\frac{1}{2}}\rho^{\frac{1}{2}}\left(Κ{\bar{C}}^{\frac{1}{2}}\right)+O\left(K^{2}\bar{C}\right)$$

where ρ is the dislocation density, A and O are the parameters determined by the effective outer cutoff radius of dislocations, b is the burgers vector, and $\bar{C}$ is the average contrast factor as explained by Ungár [17].

In a texture-free polycrystalline crystal, the contrast factor can be considered as isotropic to every reflection crystal plane, and it is also reasonable to consider all slip systems are identically populated dislocations.

The contrast factor for each slip system for BCC crystals can be calculated by:

(A3) $$\bar{C}={\bar{C}}_{h00}\left(1-qH^{2}\right)$$

where q is a parameter depending on the elastic constants of the crystal and the character of dislocation type and H is the fourth-order invariant of the hkl indices of the different reflections [40]:

(A4) $$H^{2}=\left(h^{2}k^{2}+h^{2}l^{2}+k^{2}l^{2}\right)/{\left(h^{2}+k^{2}+l^{2}\right)}^{2}$$

${\bar{C}}_{h00}$ is determined by the elastic constants and represents the average dislocation contrast factor for $\left(h00\right)$ reflection, where h=1, 2, 3, …, n. We use ${\bar{C}}_{200}$ as an example; the edge part (${\bar{C}}_{200}^{edge}=0.239$) and screw part (${\bar{C}}_{200}^{screw}=0.304$) can be separately calculated by the software named ANIZC developed by Ungár et al. [41]. Likewise, the parameter q can also be subdivided into $q^{edge}$ and $q^{screw}$; the values for α-Fe are 1.28 and 2.67, respectively [42].

Ignoring the higher-order term in Equation (A2), we can rewrite it as:

(A5) $${\left(∆K-\alpha \right)}^{2}/K^{2}={\bar{C}}_{h00}\delta^{2}\left(1-qH^{2}\right)$$

where $\alpha =\frac{0.9}{D}$ and δ = ${\left(\frac{\pi Ab^{2}}{2}\right)}^{\frac{1}{2}}\rho^{\frac{1}{2}}$. The value of q can be obtained through liner interpolation, which is related to $q^{edge}$ and $q^{screw}$ by:

(A6) $$q=hq^{edge}+\left(1-h\right)q^{screw}$$

The fraction of the edge dislocation f is:

(A7) $$f=h{\bar{C}}_{200}^{screw}/\left[{\bar{C}}_{200}^{edge}\left(1-h\right)+h{\bar{C}}_{200}^{screw}\right]$$

where h is the relative fraction of screw and edge slip systems; therefore, ${\bar{C}}_{h00}$ can be calculated by the same manner. After obtaining q and ${\bar{C}}_{h00}$, the value of $K^{2}\bar{C}$ for each diffraction peak can be determined by Equation (A3), which is an independent variable in MWH. There is a following dependence between the real part of Fourier coefficient $A\left(L\right)$ and dislocation density in MWA:

(A8) $$\ln A\left(L\right)=\ln A^{s}\left(L\right)-X\left(L\right)\left(K^{2}\bar{C}\right)+Q{\left(K^{2}\bar{C}\right)}^{2}$$

(A9) $$L=\frac{n\lambda }{2\sin\left(\theta_{2}-\theta_{1}\right)}$$

where L is the Fourier length, and n is the order of Fourier coefficients.

## Appendix B

KAM is determined by the equation:

(A10) $$\mathrm{KA}M_{i,j}=\frac{1}{\left|N\left(i,j\right)\right|}\sum_{\left(k,l\right)\in N\left(i,j\right)}\omega \left(o_{i,j},o_{k,l}\right)$$

where the $\omega \left(o_{i,j},o_{k,l}\right)$ is the disorientation angle between the current pixel and the neighboring pixel; $N\left(i,j\right)$ is the number of neighboring pixels taken into account.

The GND density can be computed based on the disorientation between pixels as well. The lattice curvature tensor is defined as:

(A11) $$k_{ij}=\left[\begin{array}{ccc} k_{11} & k_{12} & k_{13}\\ k_{21} & k_{22} & k_{23}\\ k_{31} & k_{32} & k_{33} \end{array}\right]=\frac{\partial \omega_{i}}{\partial x_{j}}≅\frac{\Delta \omega_{i}}{\Delta x_{j}}$$

where the $\Delta \omega_{i}$ are the direction cosines of the rotation axis $\vec{v}$ of the disorientation:

(A12) $$\Delta \omega_{i}=\left|\Delta \omega \right|\left(\frac{v_{i}}{\sqrt{v_{k}v_{k}}}\right)$$

Due to the fact that EBSD only measures the in-plane lattice, the disorientation angle in the third dimension is not accessible. Consequently, three components of the curvature tensor cannot be determined, i.e., $k_{13}$, $k_{23}$ and $k_{33}$. Nye’s dislocation tensor (α) is defined as:

(A13) $$\alpha =curl\varepsilon +tr\left(k\right)\cdot I-k^{T}$$

where ε is the elastic strain tensor, which can be computed by HR-EBSD patterns. Combining Equations (A11) and (A13), the Nye’s dislocation tensor can be written as:

(A14) $$\alpha_{ij}=\left[\begin{array}{ccc} \frac{{\partial \varepsilon }_{12}}{{\partial x}_{3}}-\frac{{\partial \varepsilon }_{13}}{{\partial x}_{2}} & \frac{{\partial \varepsilon }_{13}}{{\partial x}_{1}}-\frac{{\partial \varepsilon }_{11}}{{\partial x}_{3}} & \frac{{\partial \varepsilon }_{11}}{{\partial x}_{2}}-\frac{{\partial \varepsilon }_{12}}{{\partial x}_{1}}\\ \frac{{\partial \varepsilon }_{22}}{{\partial x}_{3}}-\frac{{\partial \varepsilon }_{23}}{{\partial x}_{2}} & \frac{{\partial \varepsilon }_{23}}{{\partial x}_{1}}-\frac{{\partial \varepsilon }_{21}}{{\partial x}_{3}} & \frac{{\partial \varepsilon }_{21}}{{\partial x}_{2}}-\frac{{\partial \varepsilon }_{22}}{{\partial x}_{1}}\\ \frac{{\partial \varepsilon }_{32}}{{\partial x}_{3}}-\frac{{\partial \varepsilon }_{33}}{{\partial x}_{2}} & \frac{{\partial \varepsilon }_{33}}{{\partial x}_{1}}-\frac{{\partial \varepsilon }_{31}}{{\partial x}_{3}} & \frac{{\partial \varepsilon }_{31}}{{\partial x}_{2}}-\frac{{\partial \varepsilon }_{32}}{{\partial x}_{1}} \end{array}\right]+\left[\begin{array}{ccc} -\frac{{\partial \omega }_{3}}{{\partial x}_{3}}-\frac{{\partial \omega }_{2}}{{\partial x}_{2}} & \frac{{\partial \omega }_{2}}{{\partial x}_{1}} & \frac{{\partial \omega }_{3}}{{\partial x}_{1}}\\ \frac{{\partial \omega }_{1}}{{\partial x}_{2}} & -\frac{{\partial \omega }_{1}}{{\partial x}_{1}}-\frac{{\partial \omega }_{3}}{{\partial x}_{3}} & \frac{{\partial \omega }_{3}}{{\partial x}_{2}}\\ \frac{{\partial \omega }_{1}}{{\partial x}_{3}} & \frac{{\partial \omega }_{2}}{{\partial x}_{3}} & -\frac{{\partial \omega }_{2}}{{\partial x}_{2}}-\frac{{\partial \omega }_{1}}{{\partial x}_{1}} \end{array}\right]$$

## References

[1] Dolzhenkov I.E. (1971). The nature of blue brittleness of steel. Met. Sci. Heat Treat..

[2] Caillard D. (2010). Kinetics of dislocations in pure Fe. Part I. In situ straining experiments at room temperature. Acta Mater..

[3] Caillard D., Bonneville J. (2015). Dynamic strain aging caused by a new Peierls mechanism at high-temperature in iron. Scr. Mater..

[4] Caillard D. (2016). Dynamic strain ageing in iron alloys: The shielding effect of carbon. Acta Mater..

[5] Cottrell A.H., Seeger A., Amorós J.L. Dislocations in Crystals. Proceedings of the Deformation and Flow of Solids/Verformung und Fliessen des Festkörpers, Colloquium.

[6] Chen Y., Pang J.C., Li S.X., Zou C.L., Zhang Z.F. (2022). Damage mechanism and fatigue strength prediction of compacted graphite iron with different microstructures. Int. J. Fatigue.

[7] Chen Y., Pang J.C., Zou C.L., Li S.X., Zhang Z.F. (2023). High-temperature fatigue damage mechanism and strength prediction of vermicular graphite iron. Int. J. Fatigue.

[8] Zou C.L., Pang J.C., Chen L.J., Li S.X., Zhang Z.F. (2020). The low-cycle fatigue property, damage mechanism and life prediction of compacted graphite iron: Influence of strain rate. Int. J. Fatigue.

[9] Ernould C., Beausir B., Fundenberger J.-J., Taupin V., Bouzy E., Hÿtch M., Hawkes P.W. (2022). Chapter Five—Applications of the method. Advances in Imaging and Electron Physics.

[10] Villert S., Maurice C., Wyon C., Fortunier R. (2009). Accuracy assessment of elastic strain measurement by EBSD. J. Microsc..

[11] Wilkinson A.J., Meaden G., Dingley D.J. (2006). High-resolution elastic strain measurement from electron backscatter diffraction patterns: New levels of sensitivity. Ultramicroscopy.

[12] Muránsky O., Balogh L., Tran M., Hamelin C.J., Park J.S., Daymond M.R. (2019). On the measurement of dislocations and dislocation substructures using EBSD and HRSD techniques. Acta Mater..

[13] Vershinina T., Leont’eva-Smirnova M. (2017). Dislocation density evolution in the process of high-temperature treatment and creep of EK-181 steel. Mater. Charact..

[14] Warren B.E. (1959). X-ray studies of deformed metals. Prog. Met. Phys..

[15] Warren B.E., Averbach B.L. (1950). The Effect of Cold-Work Distortion on X-ray Patterns. J. Appl. Phys..

[16] Williamson G.K., Hall W.H. (1953). X-ray line broadening from filed aluminium and wolfram. Acta Metall..

[17] Ungár T. (1998). Strain Broadening Caused by Dislocations. Mater. Sci. Forum.

[18] Ungár T. (2001). Dislocation densities, arrangements and character from X-ray diffraction experiments. Mater. Sci. Eng. A.

[19] Ashby M.F. (1970). The deformation of plastically non-homogeneous materials. Philos. Mag. A J. Theor. Exp. Appl. Phys..

[20] Fleck N.A., Ashby M.F., Hutchinson J.W. (2003). The role of geometrically necessary dislocations in giving material strengthening. Scr. Mater..

[21] Thapliyal S., Agrawal P., Agrawal P., Nene S.S., Mishra R.S., McWilliams B.A., Cho K.C. (2021). Segregation engineering of grain boundaries of a metastable Fe-Mn-Co-Cr-Si high entropy alloy with laser-powder bed fusion additive manufacturing. Acta Mater..

[22] Wan T., Cheng Z., Bu L., Lu L. (2021). Work hardening discrepancy designing to strengthening gradient nanotwinned Cu. Scr. Mater..

[23] Arsenlis A., Parks D.M. (1999). Crystallographic aspects of geometrically-necessary and statistically-stored dislocation density. Acta Mater..

[24] Pangborn R.N., Weissmann S., Kramer I.R. (1981). Dislocation distribution and prediction of fatigue damage. Metall. Trans. A.

[25] Pedrosa B., Correia J., Gripp I., Fernandes L., Rebelo C. (2023). Fatigue Life Prediction of S235 Details Based on Dislocation Density. ce/papers.

[26] (2010). High Strength Low Alloy Structural Steel Plates.

[27] (2021). Metallic Materials—Tensile Testing—Part 1: Method of Test at Room Temperature.

[28] Stokes A.R. (1948). A Numerical Fourier-analysis Method for the Correction of Widths and Shapes of Lines on X-ray Powder Photographs. Proc. Phys. Soc..

[29] Pantleon W. (2008). Resolving the geometrically necessary dislocation content by conventional electron backscattering diffraction. Scr. Mater..

[30] Bachmann F., Hielscher R., Schaeben H. (2010). Texture Analysis with MTEX–Free and Open Source Software Toolbox. Solid State Phenom..

[31] Beausir B., Fundenberger J. Analysis Tools for Electron and X-ray Diffraction. ATEX-Software, Université de Lorraine-Metz 2017. www.atex-software.eu.

[32] Hutanu R., Clapham L., Rogge R.B. (2005). Intergranular strain and texture in steel Luders bands. Acta Mater..

[33] Leslie W., Cuddy L., Sober R. (1973). Serrated yielding and flow in substitutional solid solutions of alfa iron. Proc. ICSMA3.

[34] Gubicza J. (2014). X-ray Line Profile Analysis in Materials Science.

[35] Haghshenas A., Khonsari M.M. (2018). Damage accumulation and crack initiation detection based on the evolution of surface roughness parameters. Int. J. Fatigue.

[36] Haghshenas A., Khonsari M.M. (2020). On the Recovery and Fatigue Life Extension of Stainless Steel 316 Metals by Means of Recovery Heat Treatment. Metals.

[37] Mayer T., Balogh L., Solenthaler C., Müller Gubler E., Holdsworth S.R. (2012). Dislocation density and sub-grain size evolution of 2CrMoNiWV during low cycle fatigue at elevated temperatures. Acta Mater..

[38] Zhao B., Huang P., Zhang L., Li S., Zhang Z., Yu Q. (2020). Temperature Effect on Stacking Fault Energy and Deformation Mechanisms in Titanium and Titanium-aluminium Alloy. Sci. Rep..

[39] Rui S.-S., Niu L.-S., Shi H.-J., Wei S., Tasan C.C. (2019). Diffraction-based misorientation mapping: A continuum mechanics description. J. Mech. Phys. Solids.

[40] Ungár T., Tichy G. (1999). The Effect of Dislocation Contrast on X-ray Line Profiles in Untextured Polycrystals. Phys. Status Solidi (a).

[41] Ungár T., Dragomir-Cernatescu I., Louër D., Audebrand N. (2001). Dislocations and crystallite size distribution in nanocrystalline CeO2 obtained from an ammonium cerium(IV)-nitrate solution. J. Phys. Chem. Solids.

[42] Ungár T., Schafler E., Gubicza J. (2009). Microstructure of Bulk Nanomaterials Determined by X-ray Line-Profile Analysis. Bulk Nanostructured Materials.

